# Predictors of ustekinumab intensified dose escalation in inflammatory bowel disease: a real-world (UST-predict) study

**DOI:** 10.3389/fphar.2026.1731277

**Published:** 2026-08-06

**Authors:** Mohammad Shehab, Anwar Almajdi, Israa Abdullah, Fatema Alrashed

**Affiliations:** 1 Program of Medicine, College of Medicine and Health Sciences, Abdullah Al Salem University, Khaldiya, Kuwait; 2 Division of Gastroenterology, Department of Internal Medicine, Mubarak Alkabeer, University Hospital, Jabriya, Kuwait; 3 Department of Pharmacy, The Champion Hospital, Sabah Alsalem, Kuwait; 4 Department of Pharmacy Practice, College of Pharmacy, Kuwait University, Jabriya, Kuwait

**Keywords:** Crohn’s disease, dose escalation, IBD, ulcerative colitis, ustekinumab

## Abstract

**Introduction:**

Ustekinumab is an effective biologic therapy for inflammatory bowel disease (IBD), yet many patients experience loss of response requiring dose escalation. Predictors of ustekinumab dose escalation remain poorly defined, particularly across Crohn’s disease (CD) and ulcerative colitis (UC).

**Methods:**

We conducted a retrospective cohort study to includ adult patients with IBD initiated on ustekinumab. The primary outcome was factors associated with dose escalation, defined as shortening ustekinumab maintenance interval to every 4 weeks, analyzed separately for CD and UC via multivariable logistic regression. The secondary outcome assessed predictors of time to escalation among CD patients using multivariable linear regression.

**Results:**

A total of 206 patients were included (CD: 165, UC: 41). Dose escalation occurred in 69 (41.8%) of CD and 14 (34.1%) of UC patients. In CD, prior biologic use (OR 3.05; 95% CI: 1.31–7.14; p = 0.01) and prior immunomodulator use (OR 2.07; 95% CI: 1.00–4.29; p = 0.05) were significantly associated with escalation. Longer disease duration was also observed in escalated patients (median 6 vs. 4 years, p = 0.003). In UC, none of the evaluated variables predicted escalation. Median time to escalation was 9 months (IQR 3–18) in CD and 4.5 months (IQR 2–12.8) in UC. No predictors were significantly associated with time to escalation in CD subgroup analysis.

**Conclusion:**

Prior biologic exposure, immunomodulator use, and longer disease duration predict ustekinumab dose escalation in CD but not in UC, highlighting disease-specific differences in therapeutic needs. These findings support tailored dose optimization strategies in IBD and underscore the need for prospective studies incorporating therapeutic drug monitoring and standardized escalation protocols.

## Introduction

Inflammatory bowel disease (IBD), encompassing Crohn’s disease (CD) and ulcerative colitis (UC), affects over three million adults in the United States, with incidence rates continuing to rise globally (Dahlhamer et al., 2016). The chronic, relapsing nature of these conditions significantly impacts patients’ quality of life and poses substantial healthcare costs, with annual direct medical expenses exceeding $14 billion in the United States alone (Burisch, 2025). Sustainable care strategies are needed including early biologic use, biosimilar adoption, and escalation of doses are essential to improve patient outcomes.

The advent of biologic therapies has revolutionized IBD treatment, offering targeted approaches to control inflammation and achieve sustained remission. Ustekinumab, a fully human monoclonal antibody targeting the p40 subunit shared by interleukin-12 and interleukin-23, represents a novel mechanism of action in the IBD therapeutic armamentarium (Benson et al., 2011). Initially approved for psoriasis, ustekinumab demonstrated efficacy in IBD through pivotal clinical trials, leading to regulatory approval for both Crohn’s disease and ulcerative colitis (Janssen Biotech and I nc, 2016; Alorfi et al., 2023). The standard dosing regimen consists of weight-based intravenous induction followed by subcutaneous maintenance therapy at 90 mg every 8 weeks.

Despite the proven efficacy of ustekinumab in clinical trials, real-world experience reveals that a substantial proportion of patients experience loss of response or suboptimal clinical outcomes during maintenance therapy. Studies indicate that primary non-response occurs in approximately 20%–30% of patients, while secondary loss of response affects an additional 20%–40% of initial responders over time (Ito et al., 2020). This phenomenon of diminishing therapeutic effectiveness leads to considerable clinical challenges and highlights the need for optimized dosing strategies.

Current literature demonstrates variable approaches to managing loss of response to ustekinumab, with limited standardized guidance on dose optimization strategies. While therapeutic drug monitoring has revealed correlations between serum ustekinumab concentrations and clinical outcomes, the optimal approach to dose escalation remains incompletely defined. Notably, some real-world dose escalation practices have labeled recommendations, including adjustments such as increasing ustekinumab from every 8 weeks to every 4 (Panaccione et al., 2023).

This study aims to evaluate clinical outcomes and predictive factors supporting ustekinumab dose escalation strategies in IBD patients with loss of response or suboptimal outcomes. The objectives are to assess the efficacy of shortened dosing intervals, define optimal criteria for candidate selection, and examine the safety profile of intensified ustekinumab regimens.

## Methods

### Study design

This is a retrospective single center study was conducted in a specialized gastroenterology tertiary center and performed in accordance with Strengthening the Reporting of Observational Studies in Epidemiology (STROBE) guidelines (von et al., 2008).

#### Participants

Data extraction from medical records was performed between 20th and 30th of September 2025 to include all patients with IBD who were on ustekinumab treatment between September 2019 to August 2025. The inclusion criteria were as follows: adults aged 18 years or older with a confirmed diagnosis of inflammatory bowel disease (IBD), where the diagnosis was made according to the International Classification of Diseases, 11th Revision (ICD-11, version 2025). Patients were considered to have IBD if they had ICD-11 code DD70 corresponding to Crohn’s disease (CD) or ICD-11 code DD71 corresponding to ulcerative colitis (UC). Eligible patients should have received at least one dose of ustekinumab induction IV dose and been maintained on 80 mg S.C every 8 weeks. Additionally, patients should have had moderate to severe exacerbation when started on ustkinumab defined as Crohn’s Disease Activity Index (CDAI) score of 220–450. Outcome was assessed after 8 weeks or more of receiving the first ustekinumab 80 mg S.C maintenance dose.

Exclusion criteria included patients with primary nonresponse (those who had only received IV induction and were then switched to another medication), pregnant females, patients with concomitant autoimmune diseases (such as psoriasis), and patients taking dual biologics agents (e.g., ustekinumab and another biologics). Additionally, patients with missing data and those without follow-up data were excluded.

### Data collection

Patients’ demographics and IBD related parameters were extracted from electronic medical health records. Several clinical and demographic variables were collected and examined as potential predictors of ustekinumab dose escalation and time to escalation. These included disease duration, duration of ustekinumab therapy (in months), prior use of biologic medication before initiating ustekinumab, number of prior biologic therapies, steroid use within 12 months prior to ustekinumab initiation, prior or concomitant immunomodulator use, age, sex, and inflammation (presence of at least one of the following: C-reactive protein (CRP) ≥10 mg/L, fecal calprotectin ≥250 μg/g, or albumin <35 g/L).

Ethical approval for this study was obtained on 9 October 2024 from the standing committee for the coordination of health and medical research at the ministry of health of Kuwait (IRB No. 2917/2024) and as per the updated guidelines of the Declaration of Helsinki (64th WMA General Assembly, Fortaleza, Brazil, October 2013) and the US Federal Policy for the Protection of Human Subjects.

### Outcome measures

The primary outcome of this study was to assess potential factors associated with ustekinumab dose escalation-defined as an increase in dosing frequency to every 4 weeks among patients with IBD. Outcomes were measured separately for those with CD and UC. Dose escalation status (escalated vs. not escalated) served as the dependent variable in the logistic regression analysis.

The secondary outcome was to investigate factors associated with the time to ustekinumab dose escalation among patients with CD who underwent dose intensification during the course of treatment (to every 4 weeks regimen). The time to escalation, measured in months from treatment initiation to dose adjustment, was analyzed using multivariable linear regression.

### Statistical analysis

Descriptive statistics were conducted to summarize the study variables. Categorical variables were reported as frequencies and percentages, while continuous variables were expressed as medians with interquartile ranges (IQR). To compare differences between study groups, the chi-square test was used for categorical variables, and the Mann–Whitney U test was used for continuous variables.

Stepwise multivariable logistic regression analyses were conducted separately for patients with UC and CD to identify factors associated with ustekinumab dose escalation. All logistic regression models were adjusted for age, sex, and inflammation (defined as CRP ≥10 mg/L, fecal calprotectin ≥250 μg/g, or albumin <35 g/L). Additionally, a subgroup analysis was performed among patients with CD who had dose escalation to explore potential factors influencing the time to dose escalation. Natural logarithmic transformation (log10) was applied to the dependent variable (ustekinumab dose escalation in months) to account for the skewness in the continuous time-to-escalation variable in the multivariable linear regression model.

Results of the logistic regression models are reported as odds ratios (ORs) using a null value of OR = 1. Linear regression analysis was presented as unstandardized beta coefficients (B), representing the change in the log-transformed outcome per unit increase in each predictor. To aid interpretability, the exponentiated beta values (exp[B] -or antilog[B]-) were also calculated, representing the multiplicative effect on the original (non-transformed) time to escalation. All estimates were reported with 95% confidence intervals (CIs), and a two-sided p-value of <0.05 was considered statistically significant. Statistical analyses were conducted using IBM SPSS Statistics (Version 29.0; Armonk, NY: IBM Corp.).

## Results

### Demographics: total sample

Total of 165 patients with CD were included in the analysis. The median age was 31 years (IQR 24–39), and 87 (52.7%) were male. The median disease duration prior to initiation of ustekinumab therapy was 5 years (IQR 2–11). About 53 (32.1%) of the patients were biologic-naïve, while 72 (43.6%) had received immunomodulators before starting ustekinumab therapy, and 36 (21.8%) had used steroids within 12 months prior to starting ustekinumab. A total of 69 (41.8%) of the patients underwent dose escalation during the follow-up period. The median duration of therapy since ustekinumab initiation was 43 months (IQR 23–84). Inflammatory markers included a median CRP level of 7 mg/L (IQR 3–14.5) and median fecal calprotectin of 90 μg/g (IQR 56–361.5). Serum albumin levels showed highly clustered values, with a median of 40 g/L (IQR 40–40).

UC patients were 41 in total. The median age was 30 years (IQR 25–40.5), and 22 (53.7%) were male. The median disease duration prior to starting ustekinumab was 4 years (IQR 2–7.5). A total of 18 (43.9%) of patients were biologic-naïve, 21 (51.2%) had received immunomodulators previously, and 15 (36.6%) had used steroids within 12 months prior to ustekinumab initiation. Dose escalation occurred in 14 (34.15%) of patients during follow-up. The median duration since starting ustekinumab was 34 months (IQR 12–65). Inflammatory markers included a median CRP level of 4 mg/L (IQR 2.35–15) and median fecal calprotectin of 89 μg/g (IQR 55.5–359). Serum albumin values were highly clustered, with a median of 40 g/L (IQR 40–40). All demographic information is summarized in Table 1.

**TABLE 1:** Baseline characteristics of included patients.

Variables	Total with CD n = 165	Total with UC n = 41
Age (years), median (IQR)	31 (24–39)	30 (25–40.5)
Sex, n (%) Male Female	87 (52.7) 78 (47.3)	22 (53.7) 19 (46.3)
Disease duration (years), median (IQR)	5 (2–11)	4 (2–7.5)
Biologic naïve patients, n (%)	53 (32.1)	18 (43.9)
Immunomodulator prior ustekinumab, n (%)	72 (43.6)	21 (51.2)
Steroid prior ustekinumab, n (%) *	36 (21.8)	15 (36.6)
Patients with dose escalation, n (%)	69 (41.82)	14 (34.15)
Duration ustekinumab started (months), median (IQR)	43 (23–84)	34 (12–65)
CRP, median (IQR)	7 (3–14.5)	4 (2.35–15)
Fecal calprotectin, median (IQR)	90 (56–361.5)	89 (55.5–359)
Albumin, median (IQR)^**^	40 (40–40)	40 (40–40)

N: number, IQR: interquartile range, CRP: C Reactive Protein, *Steroid use considered if within 12 months from starting UST, **Albumin shows clustered values.

Among patients with UC (Supplementary Table S1A), extensive colitis (E3) was the most prevalent disease subtype, accounting for 49% (approximately 20 patients). Left-sided colitis (E2) followed with 34% (14 patients), while ulcerative proctitis (E1) accounted for 19% (8 patients). In contrast, among patients with CD (Supplementary Table S1B), ileal subtype (L1) was the most common, observed in 52% (86 patients). Ileocolonic disease (L3) and colonic disease (L2) represented 36% (59 patients) and 10% (17 patients), respectively. Upper gastrointestinal involvement (L4) was rare, occurring in only 2% (3 patients). Perianal disease was reported in 16.8% (28 patients). Regarding disease behavior, inflammatory type (B1) was predominant at 47% (78 patients), followed by penetrating (B3) in 32% (53 patients) and stricturing (B2) in 21% (35 patients).

### Demographics per dose escalation

#### CD subgroup

Among 165 patients with CD, 69 (41.8%) required ustekinumab dose escalation to every 4 weeks. Patients who underwent dose escalation had a significantly longer disease duration compared to those without escalation (median 6 [IQR 4–13] vs. 4 [IQR 2–8] years; P = 0.003). They were also less likely to be biologic-naïve (18.8% vs. 42.7%; P = 0.005) and more likely to have prior immunomodulator use (59.4% vs. 32.3%; P = 0.001).

**FIGURE 1:**
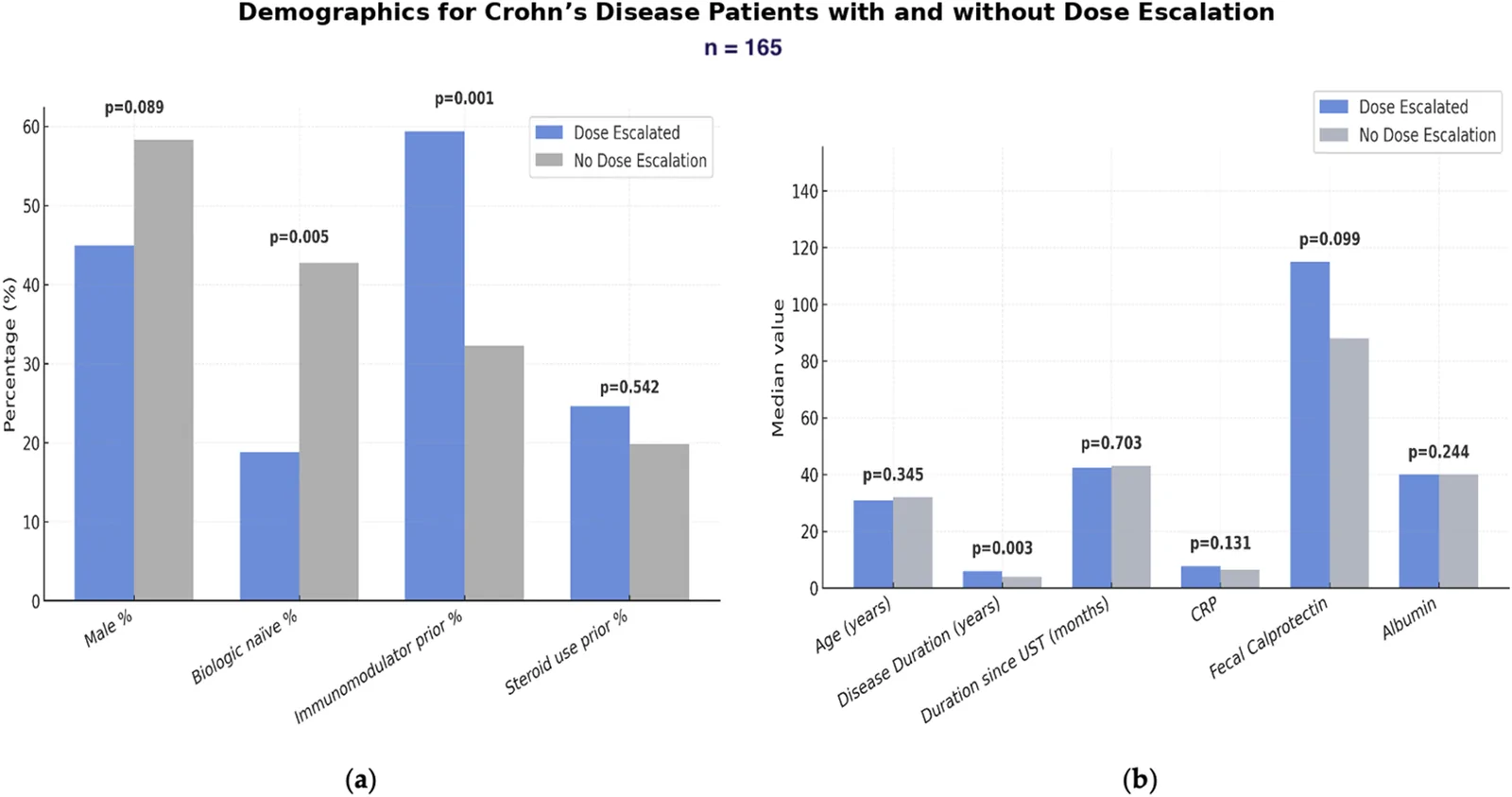
Comparative demographics for Crohn’s disease patients with and without dose escalation: **(a)** Categorical variables (percentages); **(b)** continuous variables (medians).

The median time to dose escalation was 9 months (IQR 3–18). No significant differences were observed in age, sex, steroid use, duration of ustekinumab therapy, or baseline inflammatory markers between groups (Supplementary Table S2A; Figures 1A, B).

### UC subgroup

**FIGURE 2:**
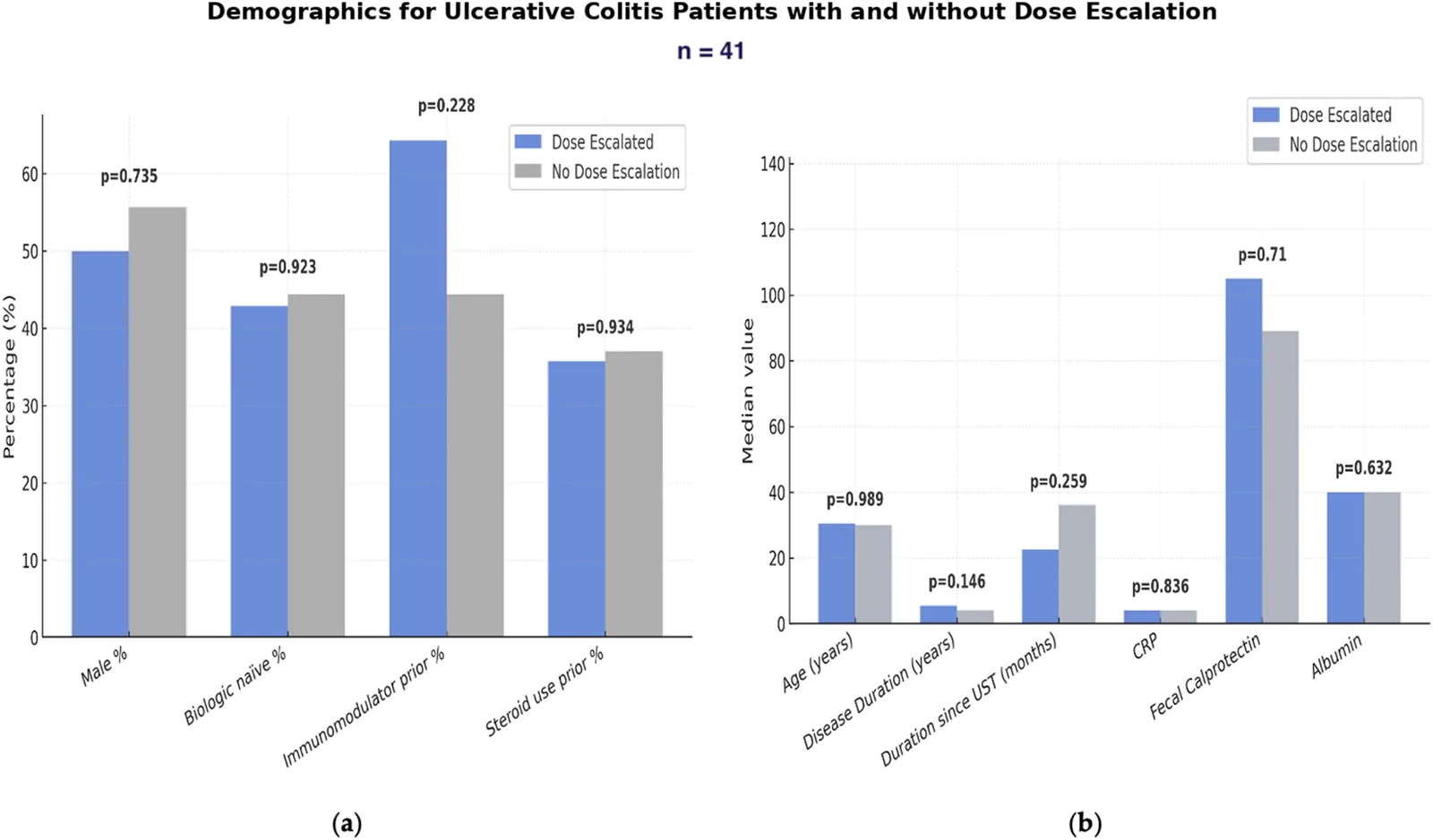
Comparative demographics for ulcerative colitis patients with and without dose escalation: **(a)** Categorical variables (percentages); **(b)** continuous variables (medians).

Among 41 patients with UC, 14 (34.1%) required dose escalation. No statistically significant differences were observed between patients with and without dose escalation in baseline demographics, disease characteristics, prior treatments, or inflammatory markers (Supplementary Table S2B; Figures 2A,B).

### Primary outcome

Among patients with CD, patients who had received prior biologic therapy were approximately three times more likely to require ustekinumab dose escalation compared to biologic-naïve patients (OR = 3.05; 95% CI: 1.31–7.14; p = 0.01). Similarly, patients with a history of immunomodulator use prior to initiating ustekinumab were twice as likely to undergo dose escalation (OR = 2.07; 95% CI: 1.00–4.29; p = 0.05). In contrast, other factors, including patient age, sex, duration of ustekinumab use, and the presence of inflammation, were not significantly associated with the likelihood of dose escalation (Supplementary Table S3). Conversely, in patients with ulcerative colitis, none of the evaluated variables, including prior biologic or immunomodulator use, were significantly associated with the likelihood of ustekinumab dose escalation (Supplementary Table S4).

### Secondary outcome

#### CD subgroup with dose escalation

**FIGURE 3:**
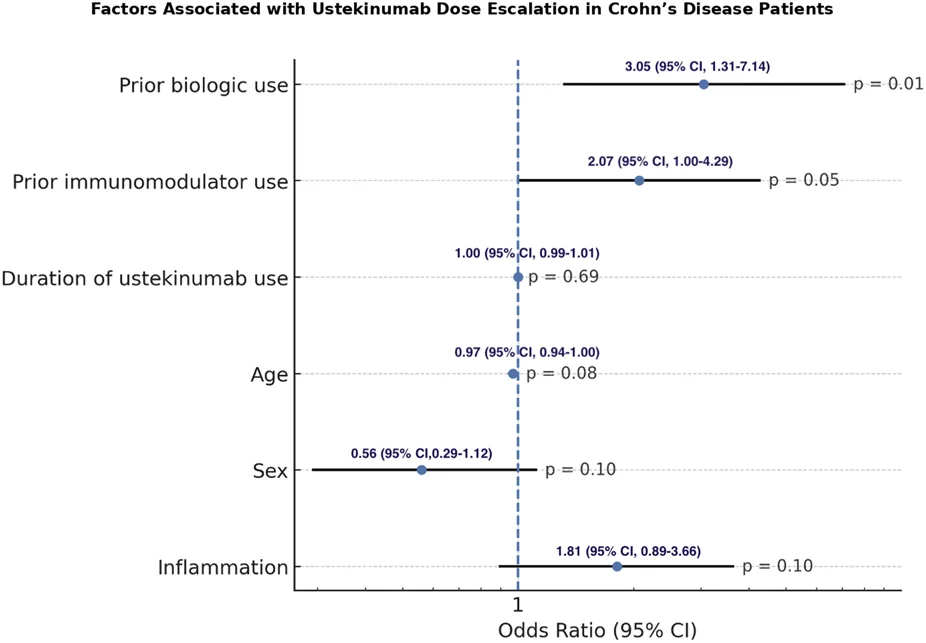
Factors associated with ustekinumab dose escalation in Crohn’s disease patients: Logistic regression model.

Post hoc analysis was conducted in the subgroup of CD patients who underwent ustekinumab dose escalation. None of the evaluated predictors were significantly associated with the time to escalation. Prior biologic use (Exp[B] = 1.82; B = 0.60; 95% CI: −6.20 to 7.40; p = 0.86) and prior immunomodulator use (Exp[B] = 0.18; B = −1.73; 95% CI: −6.86 to 3.40; p = 0.50) did not demonstrate a statistically significant effect. Similarly, the duration of ustekinumab use was not a significant predictor (Exp[B] = 1.00; B = 0.00; 95% CI: −0.06 to 0.61; p = 0.95). All models were adjusted for age, sex, and biochemical inflammation (Supplementary Table S5; Figure 3). Post hoc analysis was not conducted among patients with UC because of the small sample size.

## Discussion

This study reveals important disease-specific differences in factors predicting ustekinumab dose escalation between CD and UC patients. The primary finding demonstrates that prior biologic exposure and immunomodulator use significantly predict dose escalation in patients with CD but not in UC, highlighting the need for tailored therapeutic approaches based on disease and treatment history. Despite the availability of potent biologic therapies, improvising therapeutic strategies remains compelling today. Persistent disease activity in many patients necessitates approaches that aim to achieve deep remission while minimizing dependence on corticosteroids or surgical intervention (Lamb et al., 2019).

The findings in patients with CD show that patients with prior biologic exposure have 3-fold increased odds of requiring dose escalation (OR 3.05, p = 0.01), while those with prior immunomodulator use have a 2-fold increased risk (OR 2.07, p = 0.05). These findings suggest that prior treatment exposure is a clinically meaningful predictor of dose escalation in CD patients on ustekinumab.

The biological mechanisms underlying this association are likely multifactorial. Previous exposure to biologics may reflect a more refractory disease phenotype or alterations in inflammatory signaling pathways. Additionally, pharmacokinetic variability, including differences in drug clearance and serum ustekinumab concentrations, may contribute to reduced therapeutic response. This is supported by findings from Battat et al., who demonstrated that lower ustekinumab drug levels are associated with poorer clinical outcomes in Crohn’s disease (Battat et al., 2017). While the mechanistic basis for this association, such as prior immunological priming or altered inflammatory pathway activity requires prospective pharmacokinetic investigation, our data support the use of treatment history as a practical clinical factor when anticipating the need for dose intensification. Prior immunomodulator use may create lasting changes in immune function that affect subsequent biologic requirements, potentially through long-term immunosuppressive effects or alterations in cytokine networks (Adorini, 2003).

In contrast, patients with UC showed no significant predictors for dose escalation among the evaluated factors, which may reflect fundamental biological differences between CD and UC in their response to IL-12/23 pathway inhibition.

The 59.4% rate of prior immunomodulator use in escalated CD patients versus 32.3% in non-escalated patients (p = 0.001) represents an 84% relative increase in prior immunomodulator exposure among those requiring dose intensification. The significantly longer disease duration in dose-escalated CD patients (median 6 vs. 4 years, p = 0.003) represents a 50% longer inflammatory burden before ustekinumab initiation. The longer disease duration likely reflects cumulative structural damage, fibrosis, and altered tissues that impairs drug distribution and necessitates higher drug concentrations for therapeutic effect. Interestingly, while baseline inflammatory markers showed non-significant trends toward higher values in escalated CD patients (CRP 7.8 vs. 6.5 mg/L, p = 0.131; fecal calprotectin 115 vs. 88 μg/g, p = 0.099), these parameters were not predictive of escalation needs with ustikenumab as suggested (Gisbert and Chaparro, 2020). The median time to dose escalation of 9 months (IQR 3-18) in CD patients closely matches recent international experience, where studies report escalation timing ranging from 6 to 9 months (Rehman et al., 2025). Contrasting sharply with CD findings, UC patients showed no significant differences between escalated and non-escalated groups across all evaluated variables. The nearly identical biologic-naïve rates (42.9% vs. 44.4%, p = 0.923) and non-significant differences in prior immunomodulator use (64.3% vs. 44.4%, p = 0.228) suggest pathophysiological differences between CD and UC in their response to ustekinumab. However, the shorter median time to escalation in UC patients (4.5 vs. 9 months in CD) may reflect different inflammatory kinetics or treatment response patterns between the two diseases.

Our findings are consistent with previous real-world evidence demonstrating that ustekinumab dose escalation is frequently required in patients with IBD (Petrov et al., 2025). In a multicenter retrospective cohort, dose escalation was necessary in 55.5% of patients, with male sex identified as the only independent predictor of escalation. Importantly, dose intensification was clinically effective, resulting in steroid-free clinical remission in 66.1% of patients, normalization of C-reactive protein in 55.8%, and endoscopic healing in 35.8%. These findings support the role of ustekinumab dose escalation as an effective strategy for recapturing response in patients experiencing secondary loss of response.

A systematic review and meta-analysis of 15 real-world cohort studies included 925 patients with CD, and it demonstrated that ustekinumab dose escalation successfully recaptured clinical response in 55% of patients with inadequate response or secondary loss of response to standard dosing (Meserve et al., 2022). Additionally, 61% achieved an endoscopic response, including 29% who attained endoscopic remission. Dose interval shortening alone was effective in restoring response in 57% of patients, although no consistent predictors of successful dose escalation were identified. Collectively, these data support ustekinumab dose intensification as an effective strategy for managing loss of response in clinical practice.

The observed differences in ustekinumab dose escalation between CD and UC likely reflect fundamental differences in disease biology and treatment response. CD is characterized by transmural inflammation, greater disease heterogeneity, and involvement of any segment of the gastrointestinal tract, all of which contribute to increased inflammatory burden and pharmacokinetic variability that may necessitate dose intensification. In contrast, UC is limited to the colonic mucosa and generally exhibits a more homogeneous disease phenotype, which may result in different therapeutic requirements and response patterns to IL-12/23 inhibition. These biological differences may also explain why prior biologic exposure and immunomodulator use predicted dose escalation in CD but not in UC. Furthermore, differences in therapeutic goals and clinical decision-making may have contributed to the observed variation. Clinicians may be more inclined to escalate ustekinumab in CD to achieve transmural healing and prevent progressive structural complications, whereas in UC, persistent disease activity may more often prompt a switch to an alternative mechanism of action rather than dose intensification. Finally, the relatively small UC cohort may have limited our ability to detect significant predictors, and larger prospective studies are needed to better define factors associated with ustekinumab optimization in this population.

This study has important clinical implications for IBD management in Kuwait and similar healthcare settings. Our findings suggest that patients with CD who have previously received biologic therapy, immunomodulators, or have a longer disease duration are more likely to require ustekinumab dose escalation, allowing clinicians to identify high-risk patients early and implement closer monitoring during maintenance therapy. Recognizing these predictors may facilitate timely therapeutic optimization before significant disease progression or complications occur. In contrast, the absence of clear predictors in UC indicates that treatment decisions should rely on ongoing objective assessment rather than baseline clinical characteristics alone. Thid real-world study provide locally relevant evidence to support individualized treatment strategies and may inform future national treatment pathways and resource allocation for advanced IBD therapies.

The strengths of this study include the evaluation of both CD and UC populations in a real-world setting and the use of clinical and treatment history factors to identify predictors of escalation. Furthermore, this study provided real-world evidence that reflected everyday clinical practices and patient populations in addition to a diverse range of participants, capturing various demographics and treatment responses.

However, some limitations should be noted. The retrospective design introduces potential selection bias, and the absence of standardized escalation criteria may have influenced the results. In addition, the study was limited by sample size, particularly in the UC subgroup, which may reduce statistical power to detect predictors. Additionally, smoking was not assessed for CD patients as predictor. Finally, biochemical inflammation was defined using CRP, fecal calprotectin, and albumin, whereas decisions to escalate ustekinumab were based on a comprehensive clinical assessment incorporating symptoms, endoscopic findings, treatment response, and physician judgment. Consequently, biochemical markers alone may not have fully captured the factors driving dose escalation, and the independent association of prior biologic and immunomodulator exposure likely reflects a more treatment-refractory disease phenotype.

## Conclusion

Our study reveals that prior exposure to biologic agents, use of immunomodulators, and longer disease duration are significant predictors of ustekinumab dose escalation in Crohn’s disease. In contrast, ulcerative colitis shows no clear predictors for this phenomenon. These findings underscore the importance of disease-specific optimization strategies and suggest that proactive monitoring and early intervention could enhance long-term outcomes in IBD patients receiving ustekinumab.

## Data Availability

The raw data supporting the conclusions of this article will be made available by the authors, without undue reservation.
