# Supplementary material for: Predictors of ustekinumab intensified dose escalation in inflammatory bowel disease: a real-world (UST-predict) study
Source: Front Pharmacol. 2026 Aug 6;17:1731277. doi: 10.3389/fphar.2026.1731277 (PMC13489931; doi:10.3389/fphar.2026.1731277)
Supplement: Supplementary file 1 [file Table1.docx]

**Supplementary data**

**Supplementary Table 1A. Ulcerative Colitis Subtypes (n = 41)**

| **Subtype** | **Description** | **%** | **Number (n)** |
| --- | --- | --- | --- |
| E1 | Ulcerative proctitis | 19% | 8 |
| E2 | Left-sided colitis | 34% | 14 |
| E3 | Extensive colitis | 49% | 20 |

**Supplementary Table 1B. Crohn’s Disease Subtypes (n = 165)**

| **Subtype** | **Description** | **%** | **Number (n)** |
| --- | --- | --- | --- |
| L1 | Ileal | 52% | 86 |
| L2 | Colonic | 10% | 17 |
| L3 | Ileocolonic | 36% | 59 |
| L4 | Upper gastrointestinal | 2% | 3 |
| P | Perianal involvement | 16.8% | 28 |
| B1 | Inflammatory | 47% | 78 |
| B2 | Stricturing | 21% | 35 |
| B3 | Penetrating | 32% | 53 |

**Supplementary Table 2A.** Comparative Demographics for Crohn’s Disease Patients with and Without Dose Escalation

| **Variables** | **CDN= 165** | |  |
| --- | --- | --- | --- |
|  | Dose Escalated  N=69 (41.82%)^¥^ | No Dose  Escalation  N= 96 (58.18%) | P-Value |
| **Age (years), median (IQR)** | 31 (24-38) | 32 (25-41) | 0.345 |
| **Sex, n (%)**  **Male**  **Female** | 31 (44.9)  38 (55.1) | 56 (58.3)  40 (41.7) | 0.089 |
| **Disease Duration (years), median (IQR)** | 6 (4-13) | 4 (2-8) | 0.003 |
| **Biologic naïve patients, n (%)** | 13 (18.8) | 41 (42.7) | 0.005 |
| **Immunomodulator Prior Ustekinumab, n (%)** | 41 (59.4) | 31 (32.3) | 0.001 |
| **Steroid Use Prior Ustekinumab, n (%)*** | 17 (24.6) | 19 (19.8) | 0.542 |
| **Time to Dose Escalation (months), median (IQR)** | 9 (3-18) | _ | _ |
| **Duration Since Ustekinumab Started (months), median (IQR)** | 42.5 (20-90) | 43 (24-72) | 0.703 |
| **CRP, median (IQR)** | 7.8 (4-16) | 6.5 (3-14.8) | 0.131 |
| **Fecal Calprotectin, median (IQR)** | 115 (66-444) | 88 (50.3-346.8) | 0.099 |
| **Albumin, median (IQR)^**^** | 40 (40-40) | 40 (40-40) | 0.244 |

N: number, IQR: interquartile range, *Steroid use considered if within 12 months from starting UST,

**Albumin shows clustered values, ^¥^Presents subgroup cohort for the secondary outcome

**Supplementary Table 2B.** Comparative Demographics for Ulcerative Colitis Patients with and without Dose Escalation

| **Variables** | **UC N=41** | | |
| --- | --- | --- | --- |
|  | Dose Escalated  N= 14 (34.15%) | No Dose  Escalation  N=27 (65.85) | P-Value |
| **Age (years), median (IQR)** | 30.5 (25.5-41.8) | 30 (25-41) | 0.989 |
| **Sex, n (%)**  **Male**  **Female** | 7 (50)  7 (50) | 15 (55.6)  12 (44.4) | 0.735 |
| **Disease Duration (years), median (IQR)** | 5.5 (2.8-10.3) | 4 (2-7) | 0.146 |
| **Biologic naïve patients, n (%)** | 6 (42.9) | 12 (44.4) | 0.923 |
| **Immunomodulator Prior Ustekinumab, n (%)** | 9 (64.3) | 12 (44.4) | 0.228 |
| **Steroid Use Prior Ustekinumab, n (%) *** | 5 (35.7) | 10 (37) | 0.934 |
| **Time to Dose Escalation (months), median (IQR)** | 4.5 (2-12.8) | _ | _ |
| **Duration Since Ustekinumab (months), median (IQR)** | 22.5 (3-61) | 36 (24-67) | 0.259 |
| **CRP, median (IQR)** | 4 (2.8-22) | 4 (2-14) | 0.836 |
| **Fecal Calprotectin, median (IQR)** | 105 (55.8-389.3) | 89 (37-344) | 0.710 |
| **Albumin, median (IQR)^**^** | 40 (40-40) | 40 (40-40) | 0.632 |

^N: number, IQR: interquartile range, *Steroid use considered if within 12 months from starting UST, **Albumin shows clustered values,^

**Supplementary Table 3**. Logistic Regression model: Possible Factors Associated Ustekinumab Dose Escalation in Crohn’s Disease Patients

| **Variables** | **Odds Ratio**  **(OR)** | **95% confidence interval**  **(CI)** | **P-Value** |
| --- | --- | --- | --- |
| **Prior biologic use*** | 3.05 | 1.31-7.14 | **0.01** |
| **Prior immunomodulator use*** | 2.07 | 1.00-4.29 | **0.05** |
| **Duration of ustekinumab use** | 1.00 | 0.99-1.01 | 0.69 |
| **Age** | 0.97 | 0.94-1.00 | 0.08 |
| **Sex** | 0.56 | 0.29-1.12 | 0.10 |
| **Inflammation**** | 1.81 | 0.89-3.66 | 0.10 |

^*Previous use prior to starting ustekinumab therapy.
**CRP≥ 10, Fecal Calprotectin ≥250, or Albumin <35^

**Supplementary Table 4**. Logistic Regression model: Possible Factors Associated Ustekinumab Dose Escalation in Ulcerative Colitis Patients

| **Variables** | **Odds Ratio**  **(OR)** | **95% confidence interval**  **(CI)** | **P-Value** |
| --- | --- | --- | --- |
| **Prior biologic use*** | 0.66 | 0.14-3.26 | 0.613 |
| **Prior immunomodulator use*** | 2.37 | 0.55-10.32 | 0.249 |
| **Duration of ustekinumab use** | 0.99 | 0.97-1.02 | 0.538 |
| **Age** | 1.00 | 0.94-1.06 | 0.943 |
| **Sex** | 0.77 | 0.19-3.08 | 0.710 |
| **Inflammation**** | 0.84 | 0.18-3.94 | 0.826 |

^*Previous use prior to starting ustekinumab therapy.
**CRP≥ 10, Fecal Calprotectin ≥250, or Albumin <35^

**Supplementary Table 5**. Subgroup Secondary Outcome: Multivariable Linear Regression: Possible Predictors of Time to Dose Escalation in CD Patients on Ustekinumab^¥^

| **Variables** | **Beta**  **(B)** | **Exp(B)** | **95% confidence interval**  **(CI) for B** | **P-Value** |
| --- | --- | --- | --- | --- |
| **Prior biologic use*** | 0.60 | 1.82 | -6.20-7.40 | 0.86 |
| **Prior immunomodulator use*** | -1.73 | 0.18 | -6.86-3.40 | 0.50 |
| **Duration of ustekinumab use** | 0.00 | 1 | -.06-0.61 | 0.95 |

^*Previous use prior to starting ustekinumab therapy. Controlled for age, sex and inflammation^

^¥Subgroup analysis of patients with Crohn’s disease who experienced ustekinumab dose escalation to every 4 weeks.^
